# The diagnostic performance of quantitative mapping in breast cancer patients: a preliminary study using synthetic MRI

**DOI:** 10.1186/s40644-020-00365-4

**Published:** 2020-12-14

**Authors:** Tiebao Meng, Ni He, Haoqiang He, Kuiyuan Liu, Liangru Ke, Huiming Liu, Linchang Zhong, Chenghui Huang, Anli Yang, Chunyan Zhou, Long Qian, Chuanmiao Xie

**Affiliations:** 1 Department of Radiology, Sun Yat-sen University Cancer Center, State Key Laboratory of Oncology in South China, Collaborative Innovation Center for Cancer Medicine, Guangdong Key Laboratory of Nasopharyngeal Carcinoma Diagnosis and Therapy, Guangzhou, 510060 China; 2 Department of Thoracic Surgery, The First Affiliated Hospital of Zhengzhou University, Zhengzhou, 450052 China; 3 Department of Breast Oncology, Sun Yat-sen University Cancer Center, State Key Laboratory of Oncology in South China, Collaborative Innovation Center for Cancer Medicine, Guangdong Key Laboratory of Nasopharyngeal Carcinoma Diagnosis and Therapy, Guangzhou, 510060 China; 4 Center for MRI Research, Academy for Advanced Interdisciplinary Studies, Peking University, Beijing, 100871 China

**Keywords:** Synthetic MRI, Breast cancer, T1 mapping, T2 mapping

## Abstract

**Background:**

Previous studies have indicated that quantitative MRI (qMR) is beneficial for diagnosis of breast cancer. As a novel qMR technology, synthetic MRI (syMRI) may be advantageous by offering simultaneous generation of T1 and T2 mapping in one scan within a few minutes and without concern to the deposition of the gadolinium contrast agent in cell nucleus. In this study, the potential of quantitative mapping derived from Synthetic MRI (SyMRI) to diagnose breast cancer was investigated.

**Methods:**

From April 2018 to May 2019, a total of 87 patients with suspicious breast lesions underwent both conventional and SyMRI before treatment. The quantitative metrics derived from SyMRI, including T1 and T2 values, were measured in breast lesions. The diagnostic performance of SyMRI was evaluated with unpaired Student’s t-tests, receiver operating characteristic curve analysis and multivariate logistic regression analysis. The AUCs of quantitative values were compared using Delong test.

**Results:**

Among 77 patients who met the inclusion criteria, 48 were diagnosed with histopathological confirmed breast cancers, and the rest had benign lesions. The breast cancers showed significantly higher T1 (1611.61 ± 215.88 ms) values and lower T2 (80.93 ± 7.51 ms) values than benign lesions. The area under the ROC curve (AUC) values were 0.931 (95% CI: 0.874–0.989) and 0.883 (95% CI: 0.810–0.956) for T1 and T2 maps, respectively, in diagnostic discrimination between breast cancers and benign lesions. A slightly increased AUC of 0.978 (95% CI: 0.915–0.993) was achieved by combining those two relaxation-based quantitative metrics.

**Conclusion:**

In conclusion, our preliminary study showed that the quantitative T1 and T2 values obtained by SyMRI could distinguish effectively between benign and malignant breast lesions, and T1 relaxation time showed the highest diagnostic efficiency. Furthermore, combining the two quantitative relaxation metrics further improved their diagnostic performance.

## Background

Breast cancer is the most common malignant disease in women, and its incidence continues to increase [1, 2]. The current standard method for diagnosing breast cancer is largely dependent on MRI due to its high sensitivity [3–6]. More specifically, differential diagnosis of benign and malignant breast lesions usually requires the use of dynamic contrast-enhanced MRI (DCE-MRI), which provides information on tumor perfusion and microvessel density [7]. However, the DCE curves of breast lesions with distinct characteristics overlap, resulting in a high false positive rate and diagnostic difficulties [8]. In addition, the deposition of the gadolinium contrast agent in the cell nuclei may cause injury to the human body [9]. Furthermore, by combining the morphology, distribution, and peritumoral conditions with the enhancement pattern of the lesion, the Breast Imaging Reporting and Data System (BI-RADS®) [10] provides important guidance for the qualitative diagnosis of breast lesions and for subsequent clinical decision making. Nevertheless, there is still no effective quantitative method for the diagnosis of atypical breast lesions.

Recent study demonstrates that quantitative MRI (qMR) is beneficial for the diagnosis of breast cancer through methods such as relaxation quantitative mapping [11], magnetic resonance spectroscopy (MRS) [12], and diffusion MRI [13]. As the basic intrinsic properties of MRI physics, quantitative T1 and T2 mapping have attracted increasing attention in recent years due to their potential utility [14, 15]. Moreover, previous studies have demonstrated that quantitative MRI has the potential to diagnose breast cancer [11, 16–18]. However, the long acquisition time of conventional technologies has limited their clinical application. More importantly, there is no study reporting on the simultaneous use of qMR-based T1 and T2 measurements in the diagnosis of breast cancer. Synthetic MRI (SyMRI), a novel qMR technology, may be advantageous in that it allows the simultaneous generation of T1 and T2 maps in one scan within a few minutes, which is much shorter than the scanning time of conventional MRI [19]. It should be noted that SyMRI not only generates those MR parameters simultaneously, but also provides an intrinsic correction for B1 inhomogeneity.

Recently, SyMRI research on neurodegenerative diseases, tumors, and the musculoskeletal system has been published [19–21]. The clinical feasibility and technological reproducibility and stability of this imaging technique have also been explored [22, 23]. Regarding breast cancer, it is still unclear whether SyMRI can help improve diagnostic accuracy. Jung Y et al. [23] found that the T2 values of breast masses showed a significant positive correlation between SyMRI and traditional T2 mapping. However, other quantitative relaxation metrics, such as T1 mapping, was not investigated in that study. Hence, we hypothesize that the application of SyMRI technology in breast cancer is feasible and that quantitative maps may provide new noninvasive indicators for the differential diagnosis and prognosis of breast cancer. At the same time, we aim to test which quantitative maps achieve the best diagnostic performance in breast cancer patients.

## Methods

### Study population

**Fig. 1:**
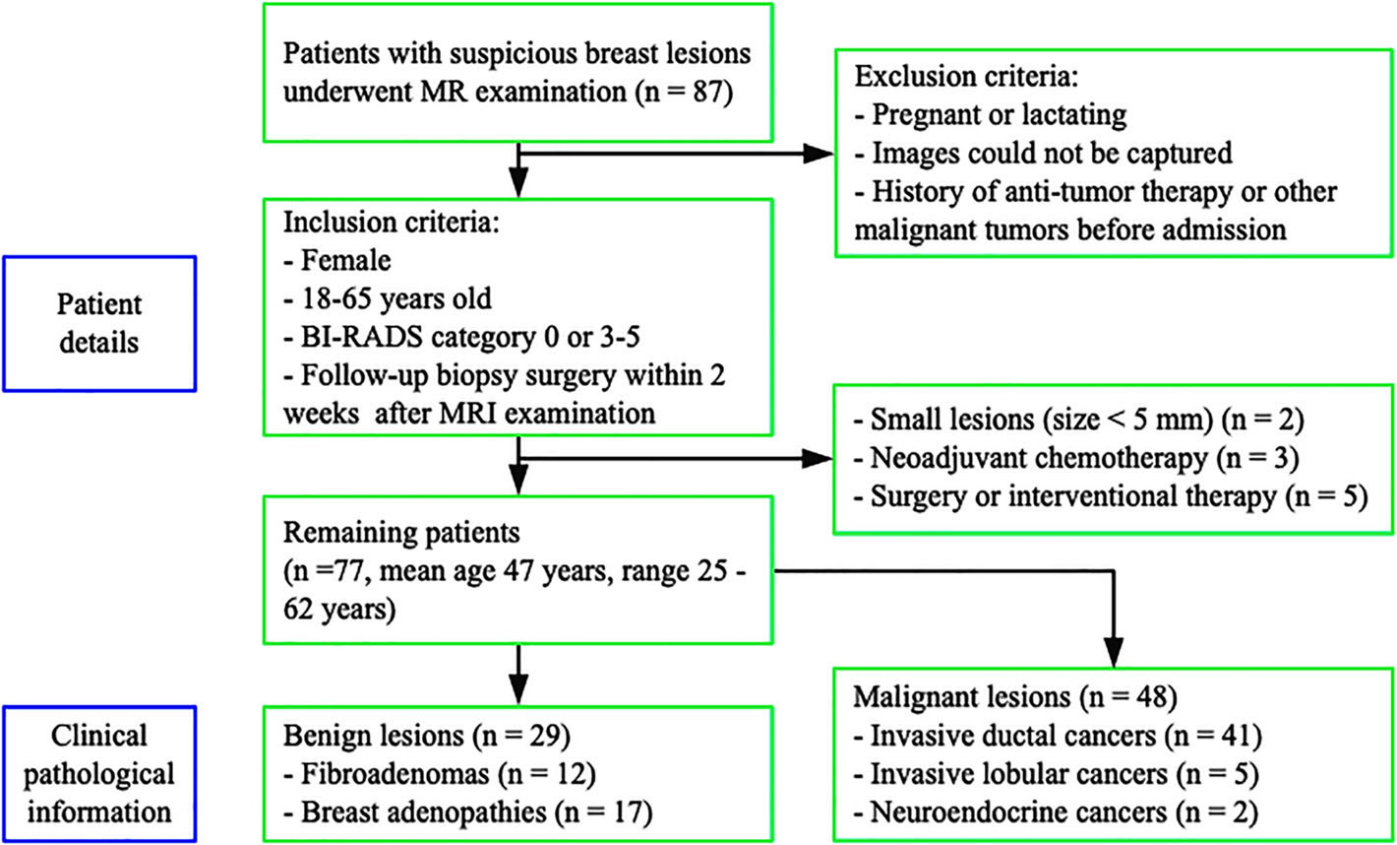
Patient details and clinical pathological information

This study is a nonrandomized, prospective, single-center research project and is approved by the Institutional Review Board. The study is also Health Insurance Portability and Accountability Act (HIPAA) compliant. Written informed consent was obtained from all participants. From April 2018 to May 2019, a total of 87 patients with suspicious breast lesions underwent MRI examination (Fig. 1). The inclusion criteria were as follows: female; 18–65 years old; BI-RADS category 0 or 3–5 at the time of initial mammography or ultrasound BI-RADS 0 or 3–5 on mammography or ultrasound; and follow-up biopsy or surgery within 2 weeks after MRI examination. The exclusion criteria were as follows: current pregnancy or lactation; history of antitumor therapy, previous history of breast cancer or other malignant tumors before admission; and inability to undergo imaging.

### MR image acquisition

All examinations were performed using a 3.0 T whole-body scanner (Signa Pioneer, GE Healthcare, WI) with an 8-channel phased-array breast surface coil. Subjects were studied in the prone position. A SyMRI sequence was added to the routine clinical MR examination. In our hospital, the regular MR imaging protocol included axial iterative decomposition of water and fat with echo asymmetry and least squares estimation T2-weighted imaging (IDEAL-T2WI), axial fast spin-echo (FSE) T1WI, axial diffusion-weighted imaging (DWI), sagittal inversion recovery T2WI in both breasts and axial three-dimensional (3D) contrast-enhanced VIBRANT-Flex. SyMRI used a 2D FSE multidelay multiecho (MDME) sequence before contrast agent injection, with the following parameters: four automatically calculated saturation delays (inversion times), recovery time (TR) = 4000 ms, echo time 1 (TE1)/echo time 2 (TE2) = 21/95 ms, slice thickness = 5 mm, interval = 1 mm, field of view (FOV) = 28 cm, image matrix = 320 × 256, receiver bandwidth = 41.67 kHz. The total scan time for SyMRI was 4 min and 30 s.

### Image analysis

The SyMRI-derived images (T1 mapping, T2 mapping, T1WI, T2WI and short-inversion-time inversion recovery (STIR)) were created using a vendor-provided program (SyMRI7.2; Synthetic MR, Linköping, Sweden). The region of interest (ROI) was placed onto the largest slice of the mass identified using the perfusion-enhanced images, avoiding the necrotic and cystic tumor areas, by two experienced radiologists with 9 and 10 years of experience, respectively. The mean T1 and T2 values were automatically calculated across all the voxels in the ROIs for each subject. The final data were reviewed by two senior radiologists.

### Data analysis

According to pathological diagnosis, the subjects were divided into two groups: benign and malignant lesions. The corresponding quantitative indicators (T1 and T2 values) of each group were tested for normality, and the mean T1 and T2 values of benign and malignant breast lesions were compared by Student’s t-test if the values were normally distributed or the Mann-Whitney U test if the distribution was non-normal. The receiver operating characteristic (ROC) curve was plotted, and the area under the ROC curve (AUC) was calculated to assess the performance of each quantitative relaxation metric in differentiating breast cancer from benign lesions. The best cutoff values for differentiating benign and malignant breast lesions were determined by maximizing the sum of sensitivity and specificity. In addition, multivariate logistic regression analysis was performed to evaluate the diagnostic performance of the combined quantitative relaxation metrics. The intraclass correlation coefficient (ICC) was used to evaluate the correlation between different observers. All of the above analyses were performed using the SPSS 25.0 statistical software package, with a value of two-tailed *p* < 0.05 considered to be a significant difference. The AUCs of quantitative values were compared using Delong test by MedCalc statistical software (www.medcalc.org).

## Results

Ten patients were excluded from the current study due to very small lesions (size < 5 mm) (*n* = 2), neoadjuvant chemotherapy (*n* = 3), and surgery or interventional therapy (*n* = 5) before MRI examination. A total of 77 lesions in 77 breasts of 77 females remained, with a mean age of 47 years (range: 25–62 years). If multiple lesions were founded in one breast, only the largest one with similar characteristics had been analyzed in one breast. All the baseline characteristics of lesions were described on Table 1.

**Table 1:** Baseline characteristics of 77 lesions^a^

	Benign		Malignant		*P -* value
Lesions	29		48		–
MRI Size (mm)	24.4 ± 10.2 (rang: 10–46)		29.5 ± 14.5 (rang: 10–66)		0.105
Age (years)	45.8 ± 7.9 (rang: 33–62)		49.1 ± 10.6 (rang: 25–62)		0.425
Hormonal Status					0.611
Premenopausal	13 (44.8%)		24 (50%)		–
Postmenopausal	16 (55.2%)		24 (50%)		–
Breast density					0.763
Non dense breast^b^	6 (20.7%)		8 (16.7%)		–
Dense breast^c^	23 (79.3%)		40 (83.3%)		–
Histopathology					–
	Fibroadenoma	12	Invasive ductal cancer	41	–
	Adenosis	17	Invasive lobular cancer	5	–
			Neuroendocrine cancer	2	

^a^Multiple lesions were founded in one breast, only the largest one with similar characteristics was analyzed in one breast

^b^Includes “almost entirely fat” and “scattered fibroglandular tissue”

^c^Includes “Heterogeneously dense” and “Extremely dense”

The ICCs between the two measurements of T1 and T2 values were identified (0.973 and 0.992, respectively). The average values based on the two reader of measurements were analyzed in this study.

**Fig. 2:**
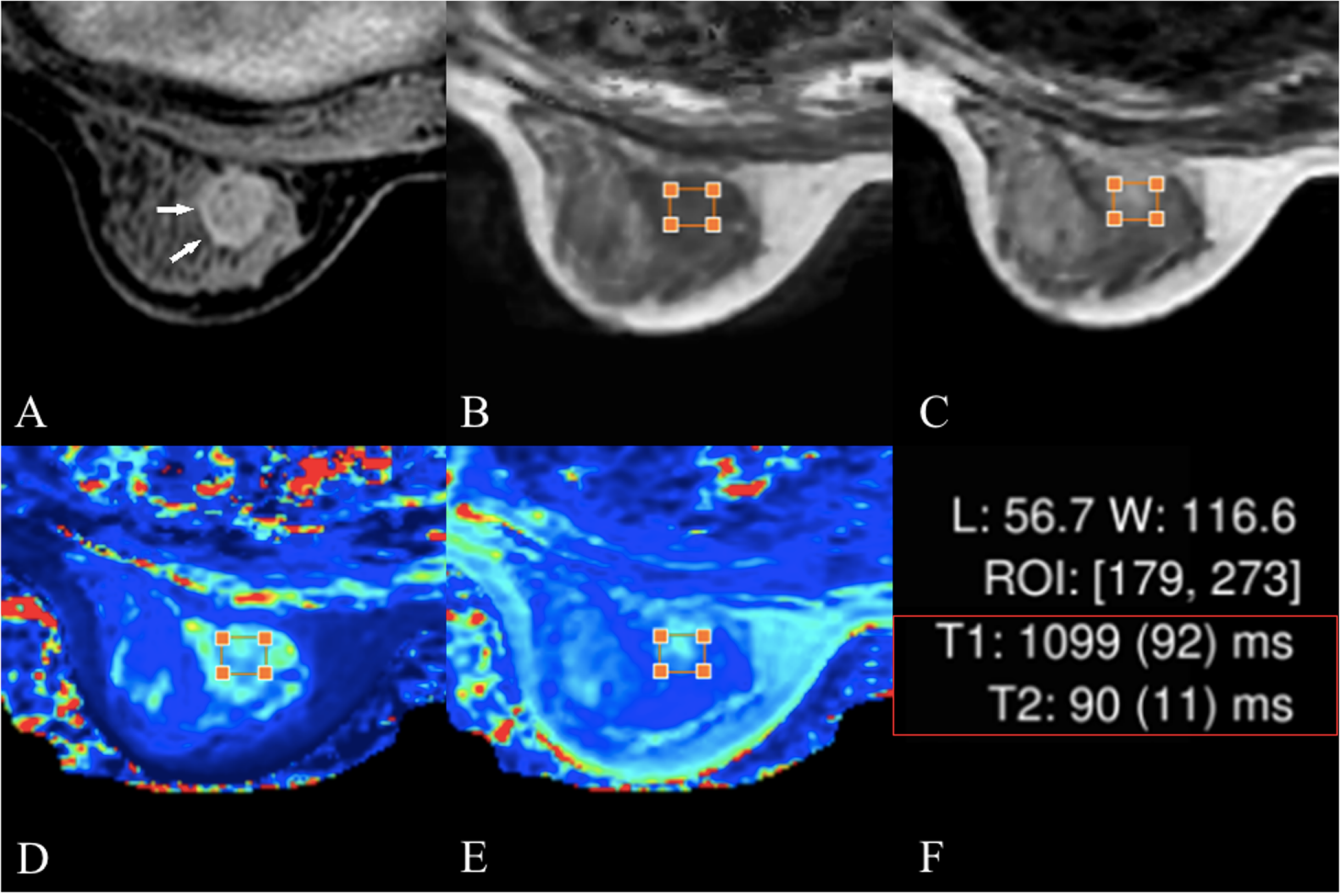
Female, 50 years old, fibroadenomas. **a** the lesion is shown using white arrows in the perfusion-enhanced image; **b** and **c**, T1-, T2-weighted images obtained from SyMR respectively I; **d** and **e** T1 and T2 maps, respectively. The T1 and T2 values are shown in **f**

**Fig. 3:**
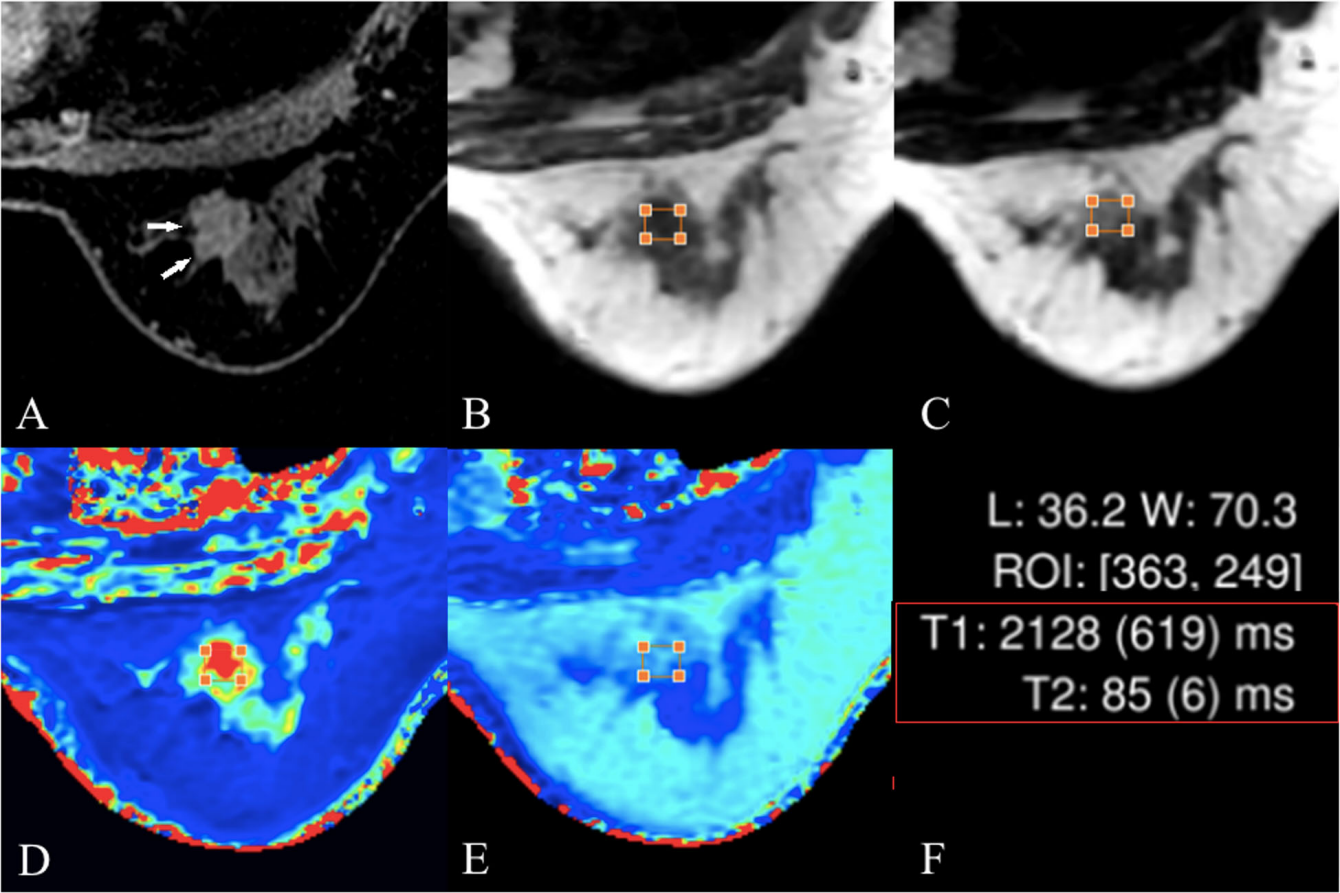
Female, 42 years old, invasive ductal cancer. **a** the lesion is shown using white arrows in the perfusion-enhanced image; **b** and **c**, T1- and T2-weighted images obtained from SyMRI respectively; **d** and **e**, T1 and T2 maps, respectively. The T1 and T2 values are shown in **f**

Details on dynamic perfusion imaging, weighted imaging and quantitative mapping in subjects with fibroadenomas and invasive carcinomas are shown in Fig. 2 and Fig. 3, respectively. According to the dynamic perfusion imaging of the breast lesions, the maximum rectangle ROI on the largest slice of the lesions was placed onto the corresponding T1W slice obtained by SyMRI. After the ROI was drawn in the SyMRI-derived image space, the quantitative features of the breast lesions could be calculated automatically (Fig. 2h and Fig. 3h).

**Fig. 4:**
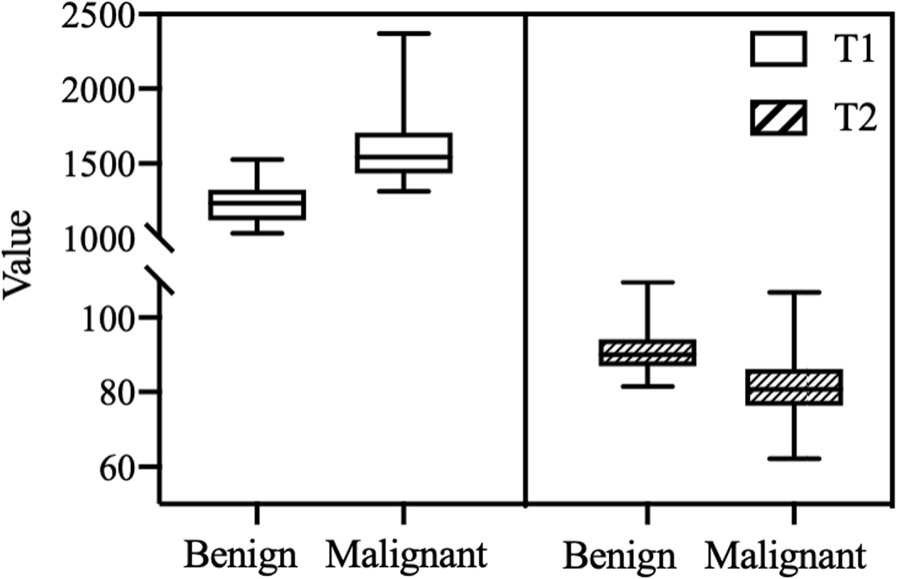
The box plots of the T1 and T2 values of benign and malignant breast lesions

The comparison of T1 and T2 values between benign and malignant breast lesions was demonstrated in Fig. 4 and Table 2. The mean T1 value of breast cancer was significantly higher than that of benign lesions (*P* < 0.001), while the mean T2 values were significantly lower than those of benign lesions (*P* < 0.001).

**Table 2:** The means and standard deviations of T1 and T2 values in benign and malignant lesions and significant *P* values (* indicates a parametric test)

	Breast lesions (*n* = 77)		*P -* value
	Benign (*n* = 29)	Malignant (*n* = 48)	
T1	1242.86 ± 139.27 ms	1611.61 ± 215.88 ms	< 0.001*
T2	91.20 ± 6.36 ms	80.93 ± 7.51 ms	< 0.001*

Note: *CI* Confidence interval

**Fig. 5:**
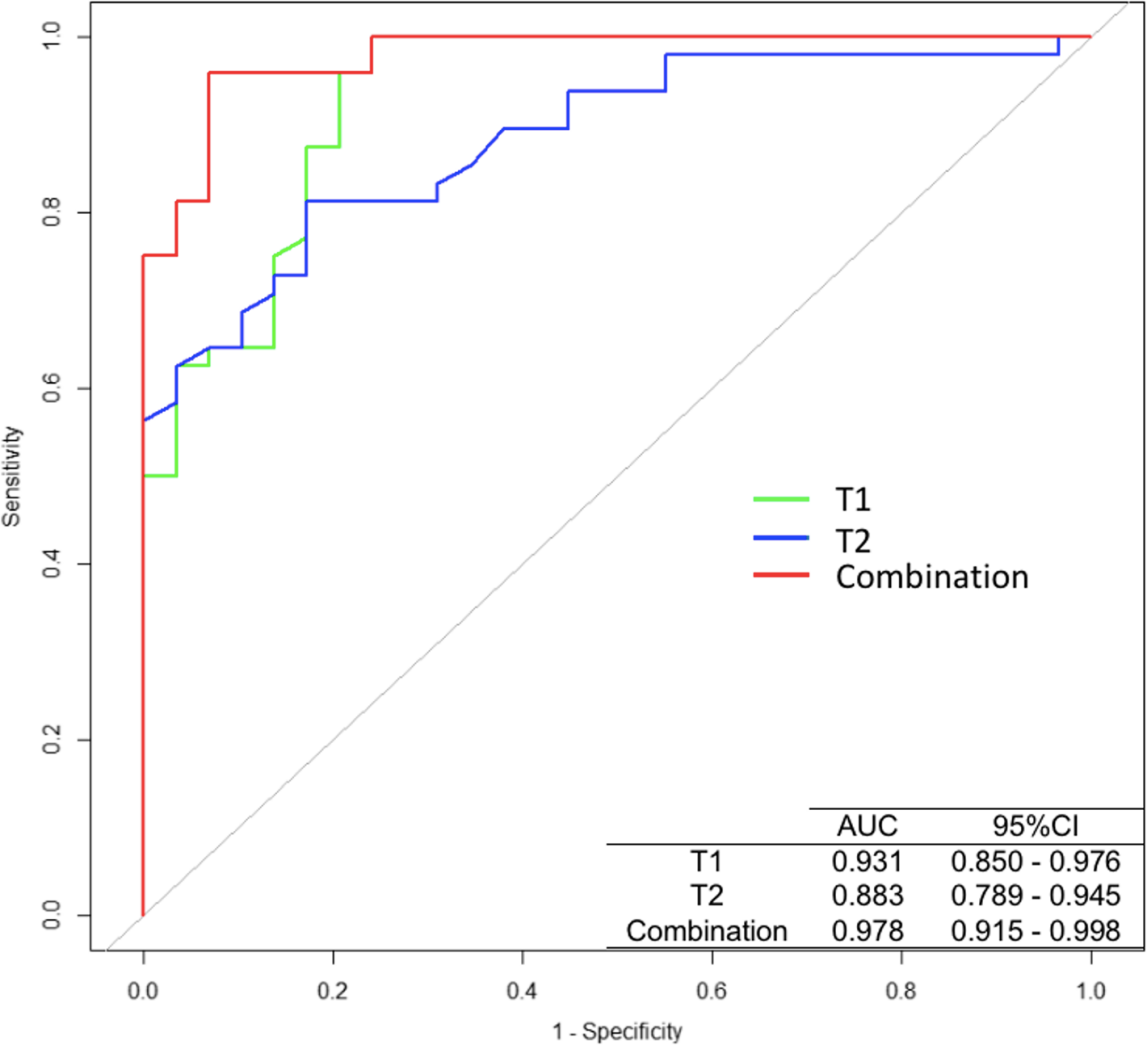
The ROC curves of T1, and T2 values and a combination of the two for the diagnosis of benign and malignant breast lesions

The ROC curves of T1, T2 and a combination of the two quantitative relaxation metrics for the diagnosis of benign and malignant breast lesions are illustrated in Fig. 5. The best cut-off value for T1 to differentiate between breast cancer and benign lesions was 1345 ms, with a sensitivity of 95.8%, a specificity of 79.3%, and an AUC of 0.931 (95% CI: 0.850–0.976). Meanwhile, the optimal cut-off value for T2 was 88.3 ms, with a sensitivity of 82.8%, a specificity of 81.3%, and an AUC of 0.883 (95% CI: 0.789–0.945). Combining the T1 and T2 values further improved their diagnostic performance in the identification of breast cancer, yielding a sensitivity of 95.8%, a specificity of 93.1%, and an AUC of 0.978 (95% CI: 0.915–0.998) (Table 3). The differences of AUCs of T1, and T2 values and combination were summarized in Table 4.

**Table 3:** The diagnostic performance of T1, and T2 values and combination of the three for breast cancer

	AUC	Cut-off value	Sensitivity (%)	Specificity (%)
T1	0.931	1345 ms	95.8	79.3
T2	0.883	88.3 ms	82.8	81.3
Combination	0.978	–	95.8	93.1

Note: *AUC* Area under curve, *CI* Confidence interval

**Table 4:** The differences of AUC of T1, and T2 values and combination

		Difference between area	95% CI	Z statistic	*P* value
T1	T2	0.0485	−0.0487 - 0.1460	0.978	0.3281
	Combination	0.0463	0.0015–0.0912	2.025	0.0429
T2	Combination	0.0948	0.0245–0.1650	2.642	0.0082

Note: *AUC* Area under curve, *CI* Confidence interval

## Discussion

SyMRI can provide 10 different contrast images and 5 quantitative maps, including T1, T2 and B1 maps, in a single scan by applying an MDME sequence [24, 25]. The application of SyMRI enables us to obtain quantitative values that cannot be obtained from conventional contrast images; this feature of SyMRI offers us an especially powerful quantitative tool in the study of disease diagnosis, efficacy evaluation and prognosis, however, it also reduces the MRI acquisition and associated potential reading time. As far as we know, this is the first time to analyze the difference of T1 and T2 values with SyMRI between benign and malignant lesions, targeted at providing more comprehensive functional characterization of breast lesions. In the current study, we found that the mean T1 value of breast cancer was significantly higher than that of benign lesions, while the mean T2 value were significantly lower than that of benign lesions. Between the two quantitative metrics, T1 mapping showed superior differential diagnostic efficiency, and combining the metrics further improved the diagnostic accuracy for breast cancer, offering important information that could improve patient care and therapy.

The T1 and T2 values depend on the composition of tissues, such as macromolecule concentration, hydration state and tissue water content [26, 27]. The components of materials are perturbed by pathophysiological conditions, and quantitative T1 and T2 values may reflect the alteration of tissue composition, indicating the nature of the possible pathological variation.

The mean T2 value of breast cancer (80.93 ± 7.51 ms) was significantly lower than that of benign lesions (91.20 ± 6.36 ms) (*P* < 0.001), as in previous studies. For example, Liu L et al. [11], using conventional T2 mapping technology, found that the mean T2 values of cancer and benign lesions were 82.69 ms and 94.48 ms, respectively. Jung Y et al. [23] also demonstrated using SyMRI that the mean T2 value of breast cancer was 84.75 ms. Those investigators noted that large breast cancer cells with abundant cytoplasm grew rapidly, resulting in a higher density of tissue cells in malignant tumors than in benign lesions. Meanwhile, cancer tissue is often accompanied by cell necrosis, and macromolecular substances are released into the space surrounding the cells, further reducing the extracellular space. There are many factors that affect breast T2 values, including water content, fibrous tissue content, and cell density of breast tissue [28]. Water in the body can be divided into free water and bound water, of which free water has a longer transverse relaxation time due to its smaller molecules. However, the binding of water by macromolecules markedly shortens its T2. Therefore, the amount of free water determines the transverse relaxation time [8]. In breast cancer, especially invasive cancer, lymphocytes and plasma cells infiltrate the interstitium, resulting in a decrease in free water. Meanwhile, in benign lesions, conditions such as myxoid degeneration in fibroadenomas and interstitial edema caused by chronic inflammation in adenopathy lengthen the transverse relaxation time of T2. Therefore, the difference in T2 value is useful for distinguishing benign lesions from breast cancers.

In addition, this study reported for the first time that T1 values were significantly different between breast cancer and benign lesions on SyMRI, with the mean T1 value of breast cancer (1611.61 ± 215.88 ms) being significantly higher than that of benign lesions (1242.86 ± 139.27 ms) (*P* < 0.001). In contrast, Chen Y et al. [29] reported a different result; they compared the T1 values of fibroglandular tissues of volunteers (1256 ± 171 ms) with that of invasive ductal cancer (1183 ± 256 ms) by qMR at 3 T and found no significant difference. The different characteristics of enrolled women in these two studies may contribute to the discrepancy. To consider the chemical shift between fat and water leading image blurring, the fat suppression modules were applied to suppress fat the signal in Chen’s study, while in our study, T1 and T2 maps of SyMRI were with fat modules, this maybe resulted in the difference of T1 values. In the cohort of young, healthy volunteers from Chen’s research, the T1 value of normal parenchyma was calculated, confirming that the parenchyma of young women was affected significantly by the hormone levels, with the T1 value fluctuating during the menstrual cycle Furthermore, more interspersed fat tissue was included in the ROIs of the healthy volunteers than in those of women with breast lesions. This difference may cause the T1 values of healthy volunteers to exceed those of patients with breast lesions. In this study, other features including age/ hormonal status breast density and the MRI size of lesions to be matched in two groups to control the bias, further research with a large sample size will help to achieve more accuracy results.

It is well known that the T1 and T2 relaxation times of tissues are independent of each other. The T1 relaxation time is the time needed to transfer the energy inside the proton group to other molecules outside; this value depends on the precession frequency of the surrounding molecules. Extracellular macromolecules correlated with cell necrosis are more plentiful in breast cancer, especially in tumors with high Ki-67 levels, than in noncancerous tissue, and the more abundant the extracellular matrix, the longer the T1 relaxation time will be, which may be the reason for the increased T1 value in breast cancer compared to benign lesions. This explanation may account for the superiority of T1 mapping over the other two measures in diagnosing breast cancer (AUC = 94%). Furthermore, T1 relaxation time is more efficient than other measures for distinguishing specific tissues [15], and in untreated tumors, lower T1 values are reportedly correlated with lower water content, higher levels of soluble protein and lower proliferation; there is also a positive correlation between T1 value of tumors and the level of Ki-67 [30]. Ki-67 is an important marker reflecting the proliferation of breast cancer cells [31].

Ni H et al. [32] confirmed that more deoxyhemoglobin (deoxyHb) was present in the cores of malignant breast tumors than in those of benign lesions. DeoxyHb has paramagnetic properties; the T2 of breast cancer is decreased because the spin of water molecules is dephased as the molecules diffuse through the local field gradients caused by the accumulation of deoxyHb, while T1 is affected by the level of deoxyHb much less than T2. Another possibility is the loss of O_2_, which acts as a relaxation agent in tissue due to the paramagnetic properties of molecular oxygen itself. In the core of a cancerous breast tumor, the amount of dissolved O_2_ will be reduced, leading to an increase in T1 [33]. Therefore, deoxyHb has a positive correlation with the T1 values of tumors and a negative correlation with their T2 values.

Although these two quantitative relaxation metrics in our study were captured before contrast administration, in the future, SyMRI may be applied both before and after contrast measurements, using with silhouettes and related techniques, to provide further information on breast lesions, but how to standardize after contrast is still a challenge.

This study had several limitations. First, we did not compare SyMRI with traditional T1 and T2 mapping, since the acquisition time for traditional T1 and T2 mapping is nearly 20 min, which may result in discomfort for patients. Second, we measured the T1 and T2 values in the largest slice of each lesion rather than the whole lesion, thus losing details from the remainder of the lesion; the values from a single slice may not represent the whole lesion. Third, future work needs to explore the SyMRI measurement in both heathy participants and participants with breast lesions, and the variation associated to different histologic classifications. Forth, the sample size in this study was small, and a larger cohort will be warranted to determine reliable ranges of T1 and T2 values for breast cancer and benign lesions.

## Conclusion

In conclusion, our preliminary study showed that the quantitative T1 and T2 values obtained by SyMRI could distinguish effectively between benign and malignant breast lesions, and T1 relaxation time showed the highest diagnostic efficiency. Furthermore, combining the two quantitative relaxation metrics further improved their diagnostic performance.

## Data Availability

All data generated or analyzed during this study are included in this published article.

## Abbreviations

SyMRI: Synthetic MRI
AUC: Area under the ROC curve
DCE-MRI: Dynamic contrast enhanced MRI
BI-RADS: Breast imaging reporting and data system
qMR: Quantitative MRI
MRS: Magnetic resonance spectroscopy
HIPAA: Health insurance portability and accountability act
IDEAL-T2WI: Iterative decomposition of water and fat with echo asymmetry and least squares estimation T2-weighted imaging
FSE: Fast spin echo
DWI: Diffusion weighted imaging
MDME: Multi-delay multi-echo
TR: Recovery time
TE: Echo time
FOV: Field of view
STIR: Short inversion time inversion recovery
ROI: Region of interest
ICC: Intraclass correlation coefficient
